# Supplementary material for: S-nitrosylation of CSF1 receptor increases the efficacy of CSF1R blockage against prostate cancer
Source: Cell Death Dis. 2022 Oct 8;13(10):859. doi: 10.1038/s41419-022-05289-4 (PMC9547886; doi:10.1038/s41419-022-05289-4)
Supplement: Supplementary file 1 — SUPPLEMENTAL MATERIAL [file 41419_2022_5289_MOESM1_ESM.docx]

**SI Appendix**

**Supp Fig 1:** (A) Graphs showing the relative expression of NOS1 and NOS2 across different Gleason Grades as studied using RNA sequencing data for Prostate adenocarcinoma from The Cancer Genome Atlas (TCGA). (B) Western blot showing the expression of NOS3 in low-grade vs. high-grade PCa.

**Supp Fig 2:** (A) Representative images for Hematoxylin and DAB staining showing expression of CSF1R and CSF1 in Normal adjacent vs. Gleason grade 9 patient biopsies. Graphs showing percent DAB intensity for relative CSF1 and CSF1R expression. (B) Representative immunohistochemical as well as Hematoxylin and eosin staining images showing expression of CD206 (M2 macrophage marker) in patient biopsies from different Gleason Grades i.e., 6, 7, and 9. (C) Immunohistochemical staining images showing expression of NOS3 and CSF1 in hormone-sensitive (LNCaP) and insensitive (H660) tumor grafts. (D) Western blot showing the expression of CSF1, NOS3, and GAPDH in LNCaP, 22RV1, and H660 tumor grafts.

**Supp Fig 3: (A)** Protein levels of AR, pERK, p90-RSK, pGSK, GSK3 BETA, VEGF and GAPDH in tumor grafts treated with CSF1R inhibitor (GW2580). (B) Representative IHC images with the quantification showing expression of AR, ARv7 and KI67 using DAB staining in tumor sections. (C) Cytokine antibody array showing selective tumor promoting candidates whose relative expression was induced or minimally changed upon CSF1R inhibition in tumor grafts. (D) Graphs showing differential immune population markers in spleenocytes.


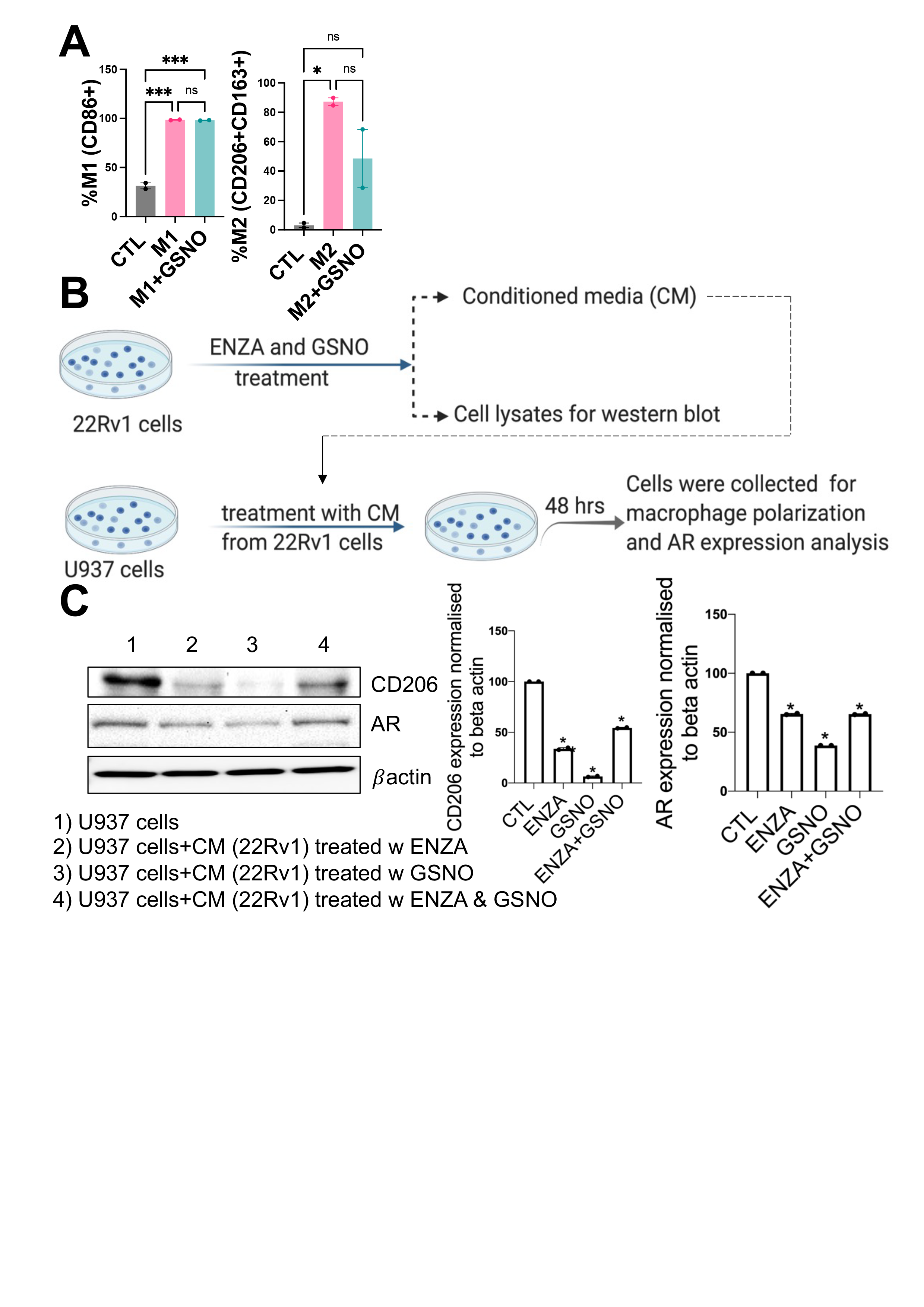
**Supp Fig 4:** (A) Percentage of M1 (CD86+) and M2 (CD206+CD163+) macrophages in U937 cells used as a model for macrophage differentiation using specific M1 and M2 cocktails when these cells were treated in the presence/absence of GSNO. (B) Experimental plan showing the course of treatment in U937 cells when these are exposed to conditioned media collected from 22Rv1 cells exposed to AR antagonist (Enzalutamide) with/without GSNO. (C) Western blot analysis and quantification for AR and CD206 expression done in U937 cells treated with different treatment conditions.

**Supp Fig 5:** Representative IHC images as well as quants for AR, ARv7 and Ki67 expression as estimated in tumor sections treated with vehicle and GSNO.


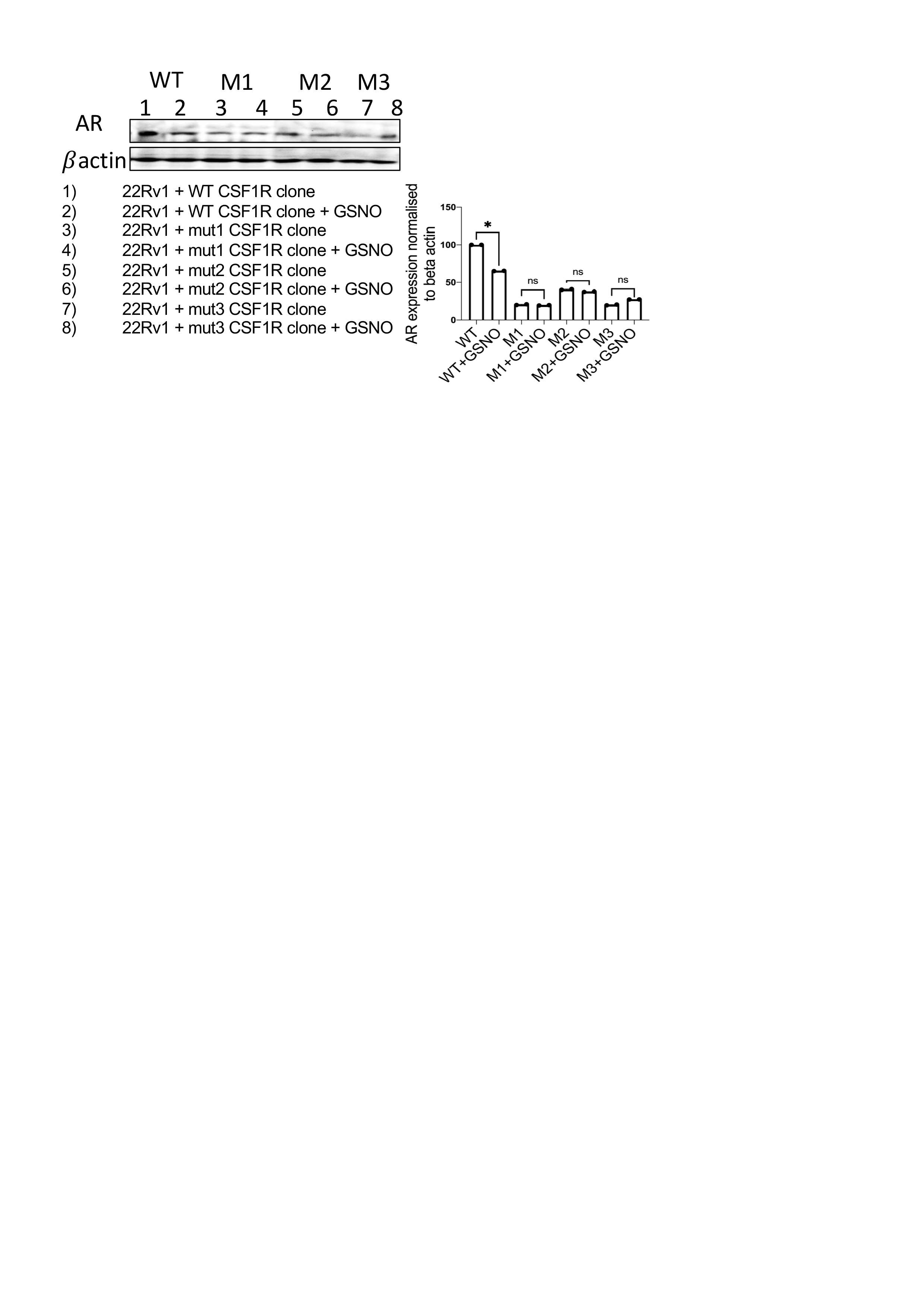


**Supp Fig 6:** Analysis of AR expression in 22Rv1 cells transfected with WT and 3 mutants in the presence and absence of GSNO.

**
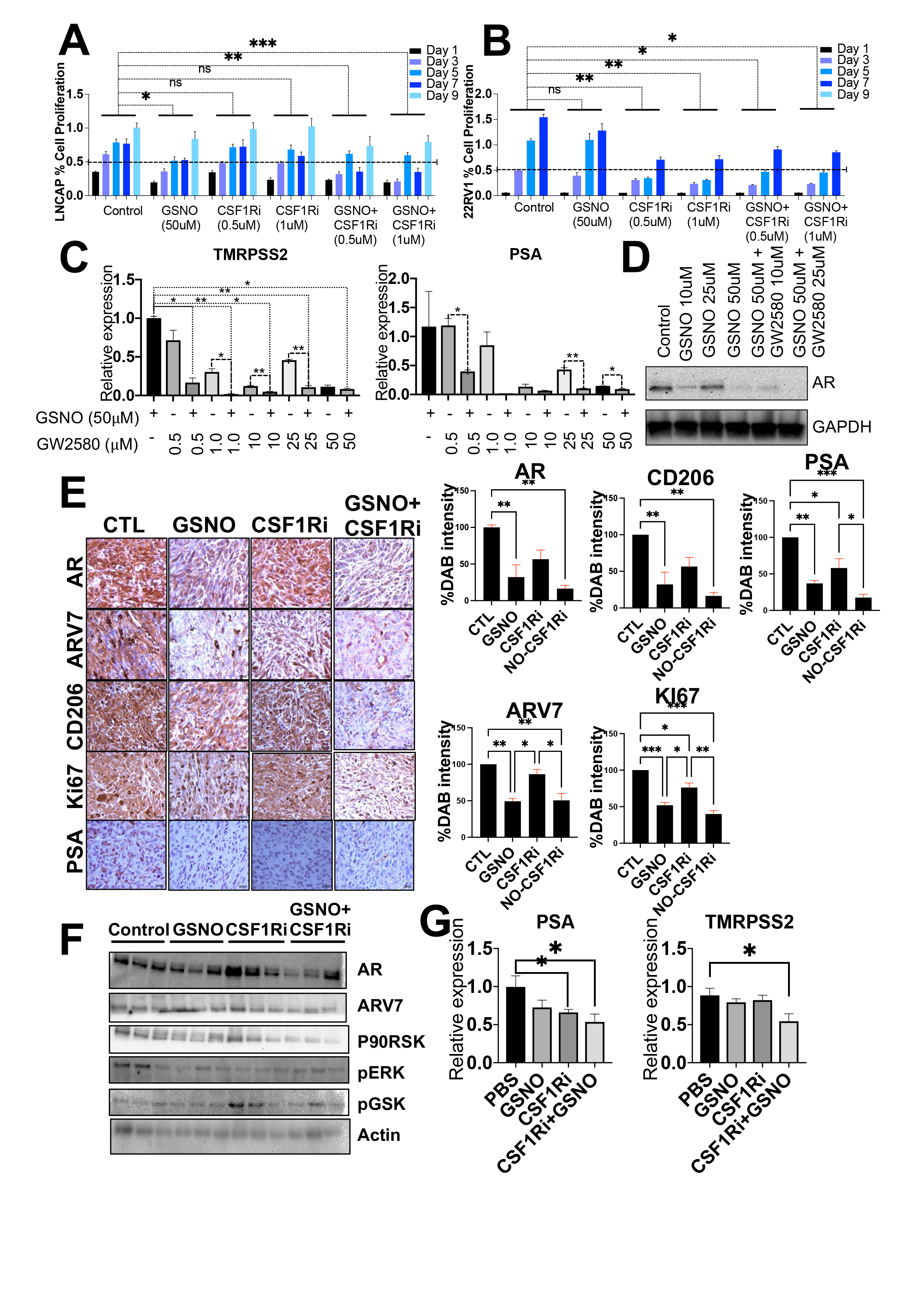
Supp Fig 7:** (A) Impact of increasing concentrations of GW2580 and fixed concentration of GSNO (50uM) evaluated on LNCAP and (B) 22RV1 cell proliferation using MTT assay (C) RNA levels of TMRPSS2 and PSA under different treatment conditions. (D) Protein levels of AR in 22Rv1 cells treated with variable doses of GSNO and CSF1R inhibitor (GW2580). (E) Western blot analysis for checking the levels of AR, ARv7, p90RSK, pERK, and pGSK in tumor grafts treated with vehicle, GSNO (10 mg/kg), GW2580 (40 mg/kg) and a combination of both GSNO and GW2580 respectively (n=3). Beta actin was used as a loading control. (F) mRNA levels of PSA and TMPRSS2 estimated in tumor grafts. Data is presented as mean ± SEM.


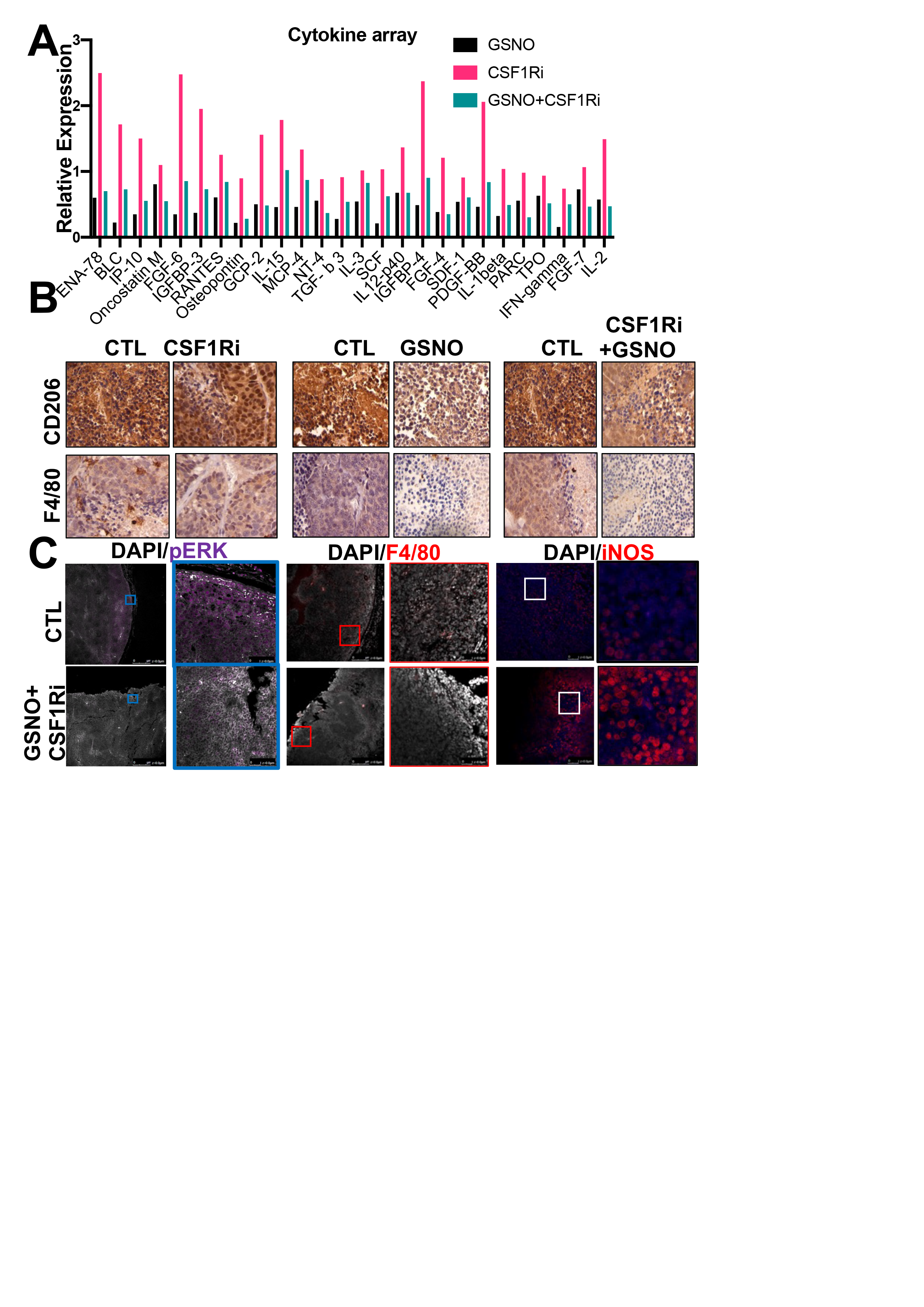


**Supp Fig 8:** (A) ) Cytokine antibody array showing selective tumor promoting candidates whose relative expression was reduced/induced upon GSNO, CSF1R as well as combination treatment as estimated in 22Rv1 tumor grafts. (B) Immunohistochemistry images showing relative expression using DAB staining for the expression of AR, ARv7, CD206, KI-67, and PSA in TRAMPC2 mice exposed to GSNO, CSF1Ri, and NO-CSF1Ri treatments. (C) Immunohistochemical images showing the expression of M2 macrophage markers-CD206 and F4/80 in 22Rv1 tumors (D) Representative immunofluorescence images showing the impact of NO-CSF1Ri treatment on the expression of pERK, F4/80, and iNOS in 22Rv1 xenografts.

**Supplementary Table 1**: Table showing list of cytokines involved in various cancers and their role in modulating tumor microenvironment.

**Supplementary Table 2**. Shows antibodies that were used in immunophenotypic panel to study the impact of treatments on several immune cells.

**Supplementary Table 3**. Shows potential cystine sites on CSF1R which could be S-nitrosylated by NO. A total of 20 cystine sites were identified and the information of predicted threshold for being S-nitrosylates shown.

**KEY RESOURCE TABLE**

| **REAGENT or RESOURCE** | **SOURCE** | | **IDENTIFIER** |
| --- | --- | --- | --- |
| **Antibodies** |  |  |  |
| Anti-Ly6G ab | ThermoFisher | | Cat # 15-9668-82 |
| Anti- CD19 ab | ThermoFisher | | Cat # 35-0193-82 |
| Anti-iNOS ab | ThermoFisher | | Cat # 25-5920-82 |
| Anti-Arg1 ab | ThermoFisher | | Cat # 17-3697-82 |
| Anti-MHCII ab | ThermoFisher | | Cat # 58-5321-82 |
| Anti- FOXP3 ab | ThermoFisher | | Cat # 12-5773-82 |
| Anti-Ki67 ab | ThermoFisher | | Cat # 47-5698-82 |
| Anti- Ly6C / Ly6G ab | BD Biosciences | | Cat # 741226 |
| Anti-TCRB ab | BD Biosciences | | Cat # 749914 |
| Anti-CD45 ab | BD Biosciences | | Cat # 741957 |
| Anti-CD62L ab | BD Biosciences | | Cat # 741230 |
| Anti-CD44 ab | BD Biosciences | | Cat # 612799 |
| Anti-PD-L1 ab | Biolegend | | Cat # 124315 |
| Anti-CD206 ab | Biolegend | | Cat # 141723 |
| Anti-CD11b ab | Biolegend | | Cat # 101267 |
| Anti- F4/80 ab | Biolegend | | Cat # 123120 |
| Anti- CCR7 ab | Biolegend | | Cat # 120106 |
| Anti- CD8 ab | Biolegend | | Cat # 100752 |
| Anti- PD-1 ab | Biolegend | | Cat # 101267 |
| Anti-GAPDH (14C10) Rabbit mAb | Cell Signaling Technology | | Cat#2118 |
| Anti-eNOS (49G3) Rabbit mAb | Cell Signaling Technology | | Cat#9586S |
| Recomb Anti-CSF1R Rabbit monoclonal Ab | Abcam | | Cat#ab271294 |
| Recomb Anti-M-CSF Rabbit monoclonal Ab | Abcam | | Cat#ab233387 |
| Anti-Cysteine Sulfenic Acid Rabbit polyclonal Ab | Sigma Aldrich | | Cat#07-2139-I |
| Anti-eNOS mouse monoclonal Ab | Abcam | | Cat# ab76198 |
| Normal mouse IgG | Santa Cruz | | Cat#SC-2025 |
| Normal Rabbit IgG | Santa Cruz | | Cat# SC-2027 |
| Anti-β-Actin (AC-15) Mouse mAb | Sigma | | Cat#A1978; RRID:AB_476692 |
| Anti-Mouse IgG (H+L), HRP Conjugate | Promega | | Cat#W4021; RRID:AB_430834 |
| Anti-Rabbit IgG (H+L), HRP Conjugate | Promega | | Cat#W4011; RRID:AB_430833 |
| **Biological Samples** |  | |  |
| Prostate Biopsies | University of Miami | |  |
| **Chemicals, Peptides, and Recombinant Proteins** |  | |  |
| Collagenase from Clostridium histolyticum Type IA | Sigma-Aldrich | |  |
| TRIzol Reagent | Invitrogen | | Cat#C9891 |
| iQ™ SYBR® Green Supermix | Bio-Rad | Cat#10296-028 | |
| RIPA Buffer | Cell Signaling | | Cat# PHG0026 |
| PhosphataseArrest™ Phosphatase Inhibitor Cocktail | G-Biosciences | | Cat#9806 |
| ProteaseArrest™ Protease Inhibitor Cocktail | G-Biosciences | | Cat#786-450 |
| Pierce™ ECL Western Blotting Substrate | Thermo Scientific | | Cat#786-331 |
| **Critical Commercial Assays** |  | | Cat#32106 |
| iScript cDNA Synthesis Kit | Bio-Rad | |  |
| RayBio Cytokine antibody Array Kit | RayBio | |  |

| **Software and Algorithms** | | |
| --- | --- | --- |
| Adobe Illustrator | Adobe Systems, San Jose, CA | <https://www.adobe.com/ca/products/illustrator.html> |
|  |  |  |
| FlowJo software V10 | FlowJo, LLC | https://www.flowjo.com/ |
