# Supplementary material for: S-nitrosylation of CSF1 receptor increases the efficacy of CSF1R blockage against prostate cancer
Source: Cell Death Dis. 2022 Oct 8;13(10):859. doi: 10.1038/s41419-022-05289-4 (PMC9547886; doi:10.1038/s41419-022-05289-4)
Supplement: Supplementary file 2 — Uncut blots [file 41419_2022_5289_MOESM2_ESM.pdf]

Fig 1d

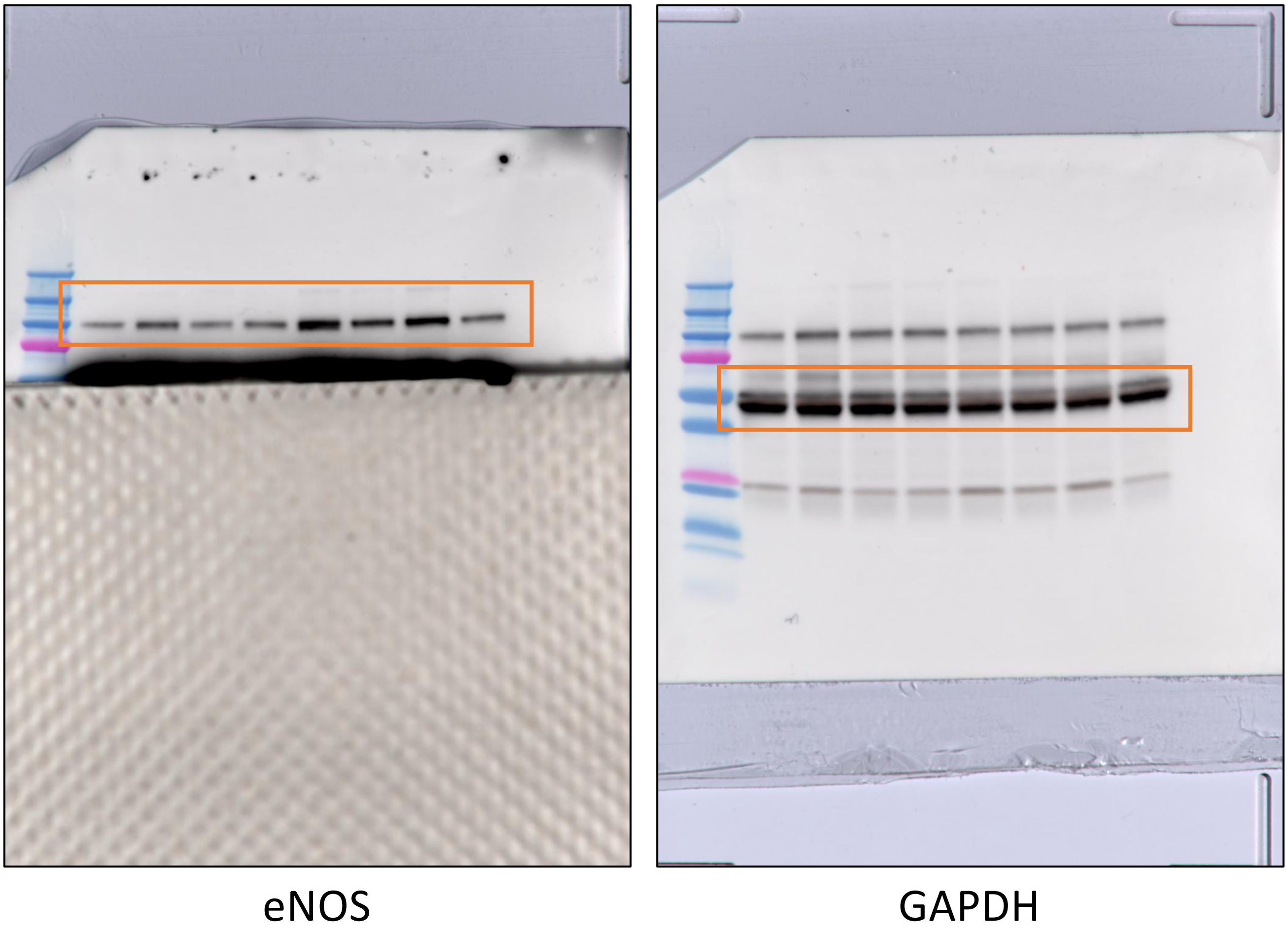

Fig 3D

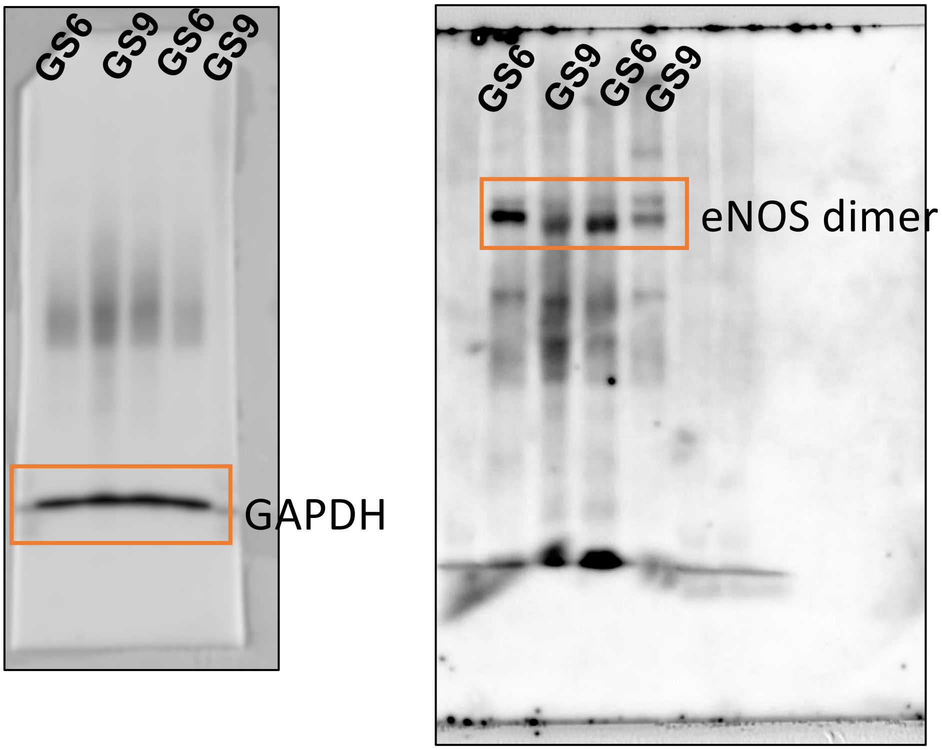

Fig 6A

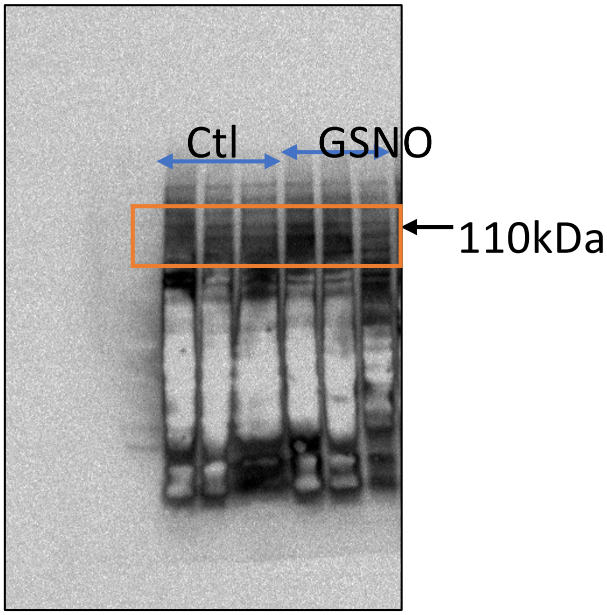

Fig 6D

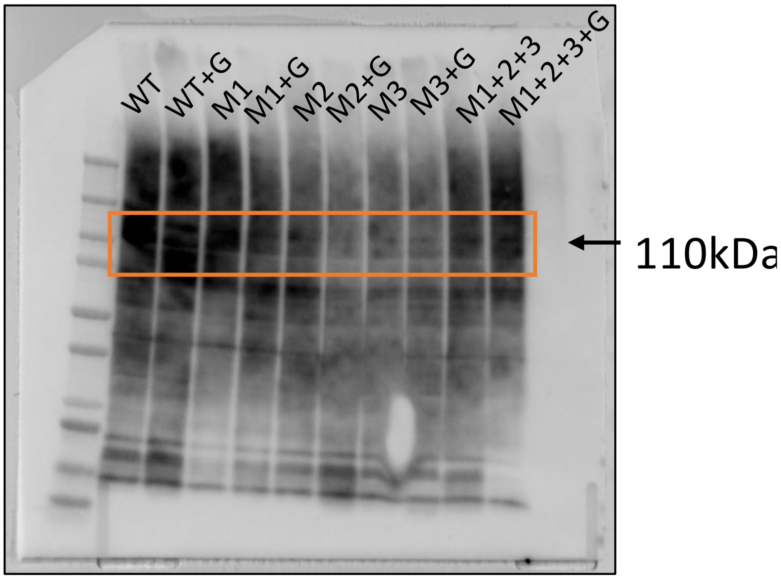

Supp Fig 1B

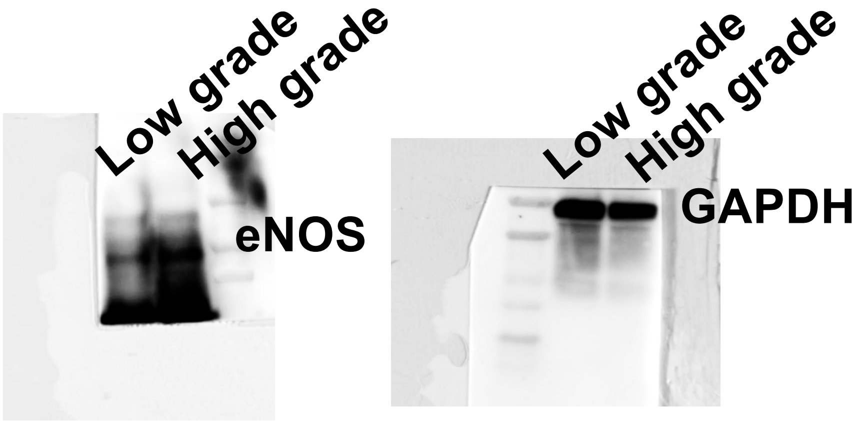

Supp Fig 2D

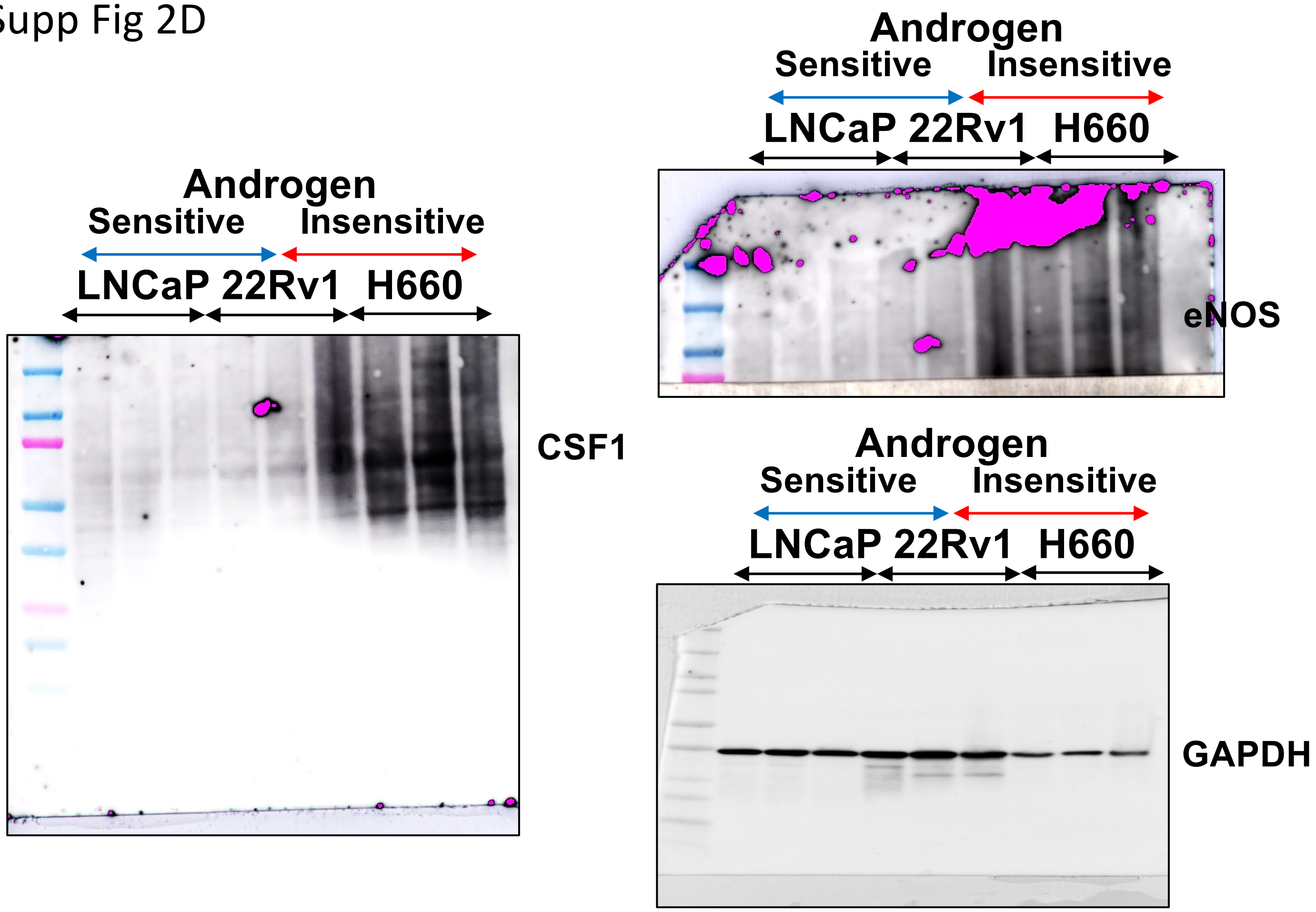

Supp Fig 3A

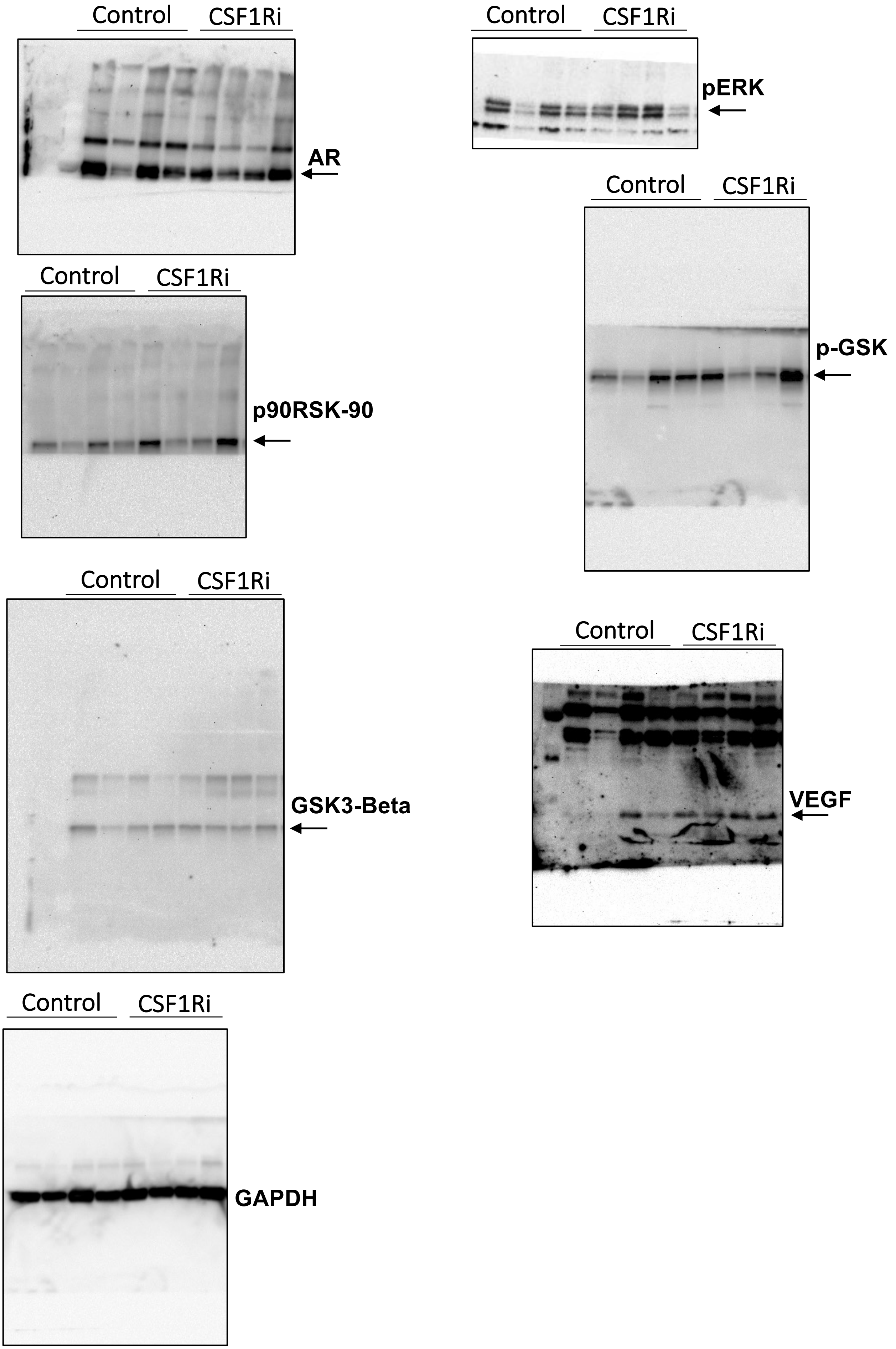

Control
CSF1Ri
AR
Control
CSF1Ri
pERK
Control
CSF1Ri
p90RSK-90
Control
CSF1Ri
p-GSK
Control
CSF1Ri
GSK3-Beta
Control
CSF1Ri
VEGF
Control
CSF1Ri
GAPDH

Sup Figure 4C

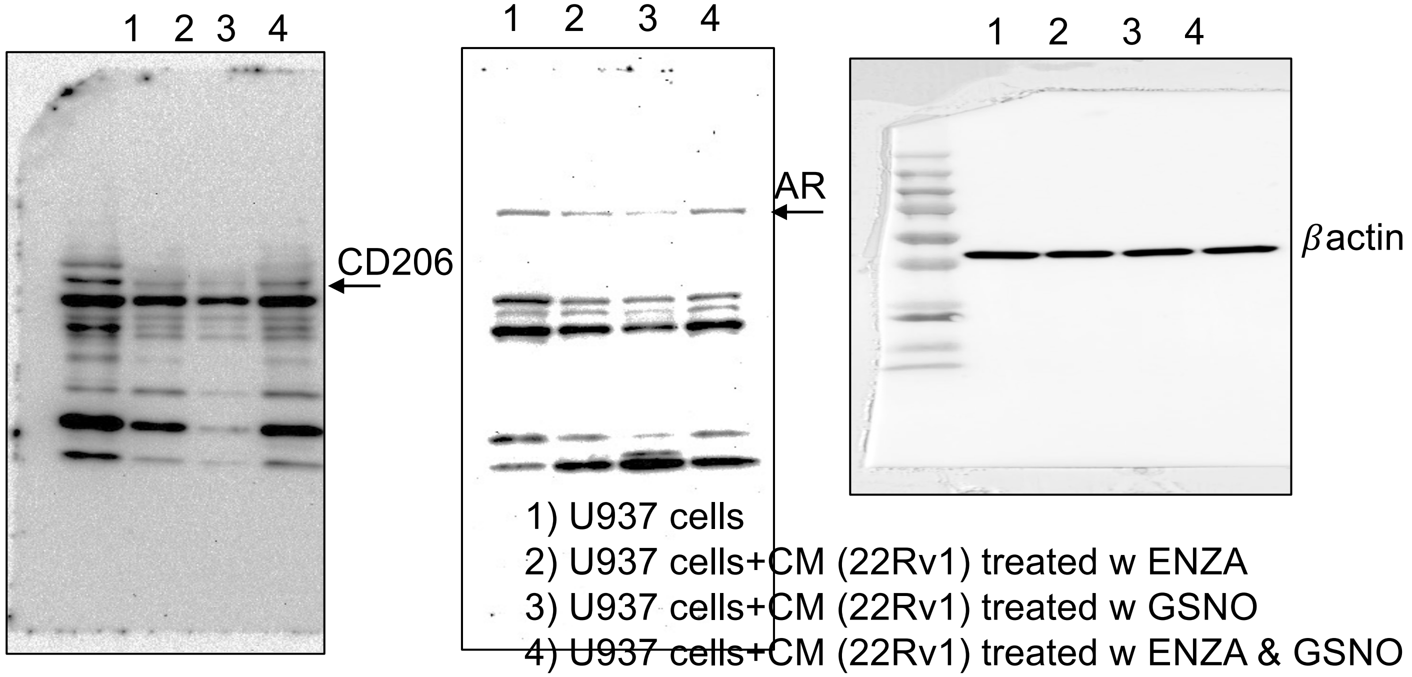

Sup Fig 6B

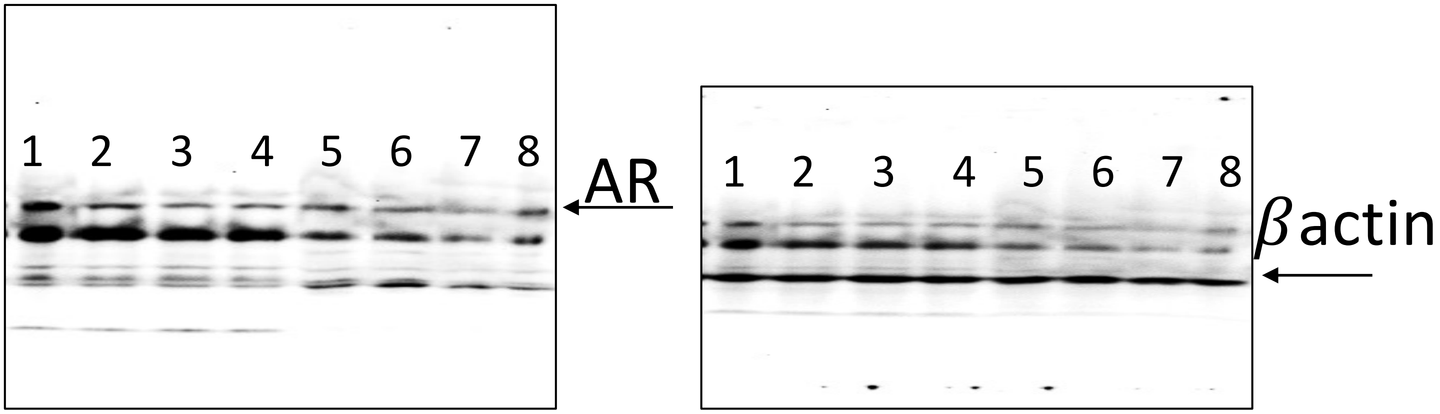

1) 22Rv1 + WT CSF1R clone
2) 22Rv1 + WT CSF1R clone + GSNO
3) 22Rv1 + mut1 CSF1R clone
4) 22Rv1 + mut1 CSF1R clone + GSNO
5) 22Rv1 + mut2 CSF1R clone
6) 22Rv1 + mut2 CSF1R clone + GSNO
7) 22Rv1 + mut3 CSF1R clone
8) 22Rv1 + mut3 CSF1R clone + GSNO

Supp Fig 7D

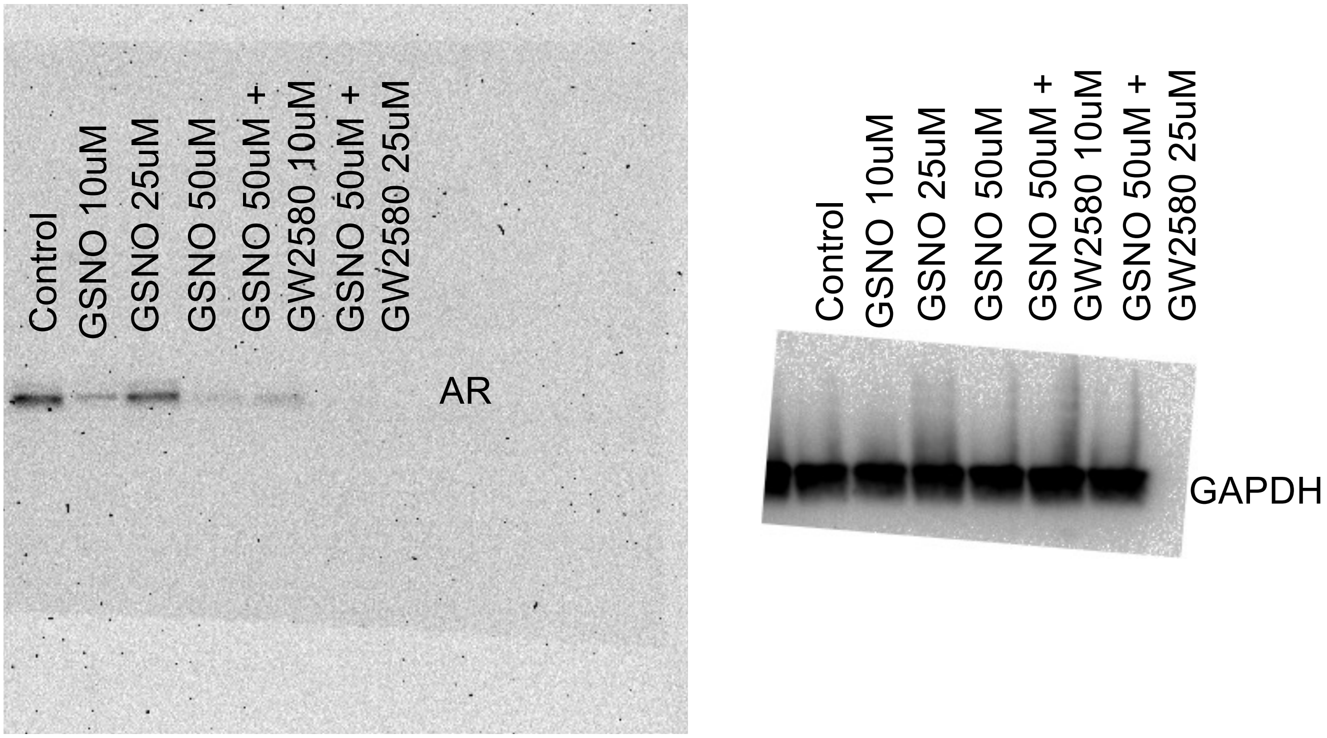

Supp Fig 7F

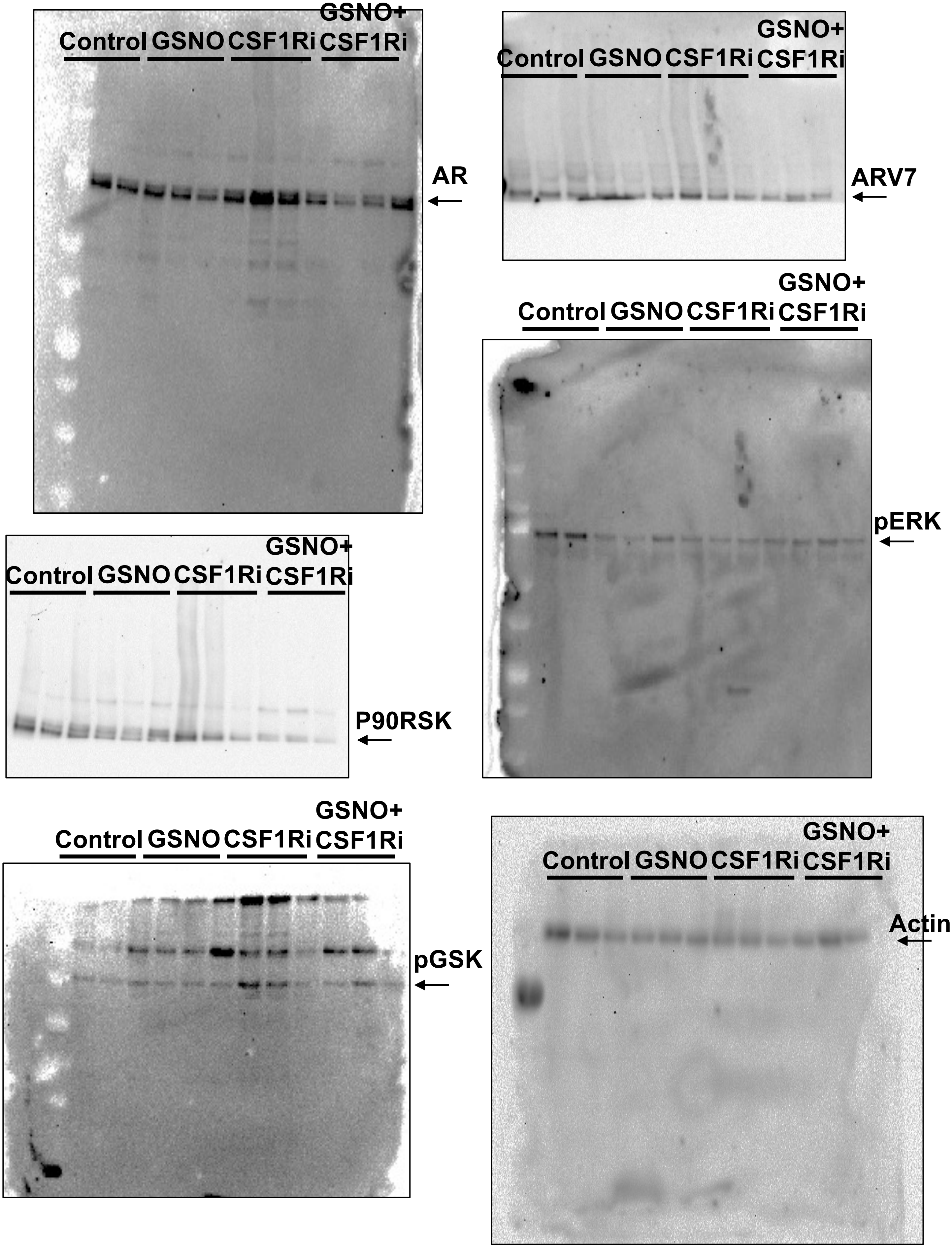
Control GSNO CSF1Ri GSNO+ CSF1Ri
AR
Control GSNO CSF1Ri GSNO+ CSF1Ri
ARV7
Control GSNO CSF1Ri GSNO+ CSF1Ri
pERK
Control GSNO CSF1Ri GSNO+ CSF1Ri
P90RSK
Control GSNO CSF1Ri GSNO+ CSF1Ri
pGSK
Control GSNO CSF1Ri GSNO+ CSF1Ri
Actin
